# Assessing Right Ventricular Function in Patients with Hypertrophic Cardiomyopathy with Cardiac MRI: Correlation with the New York Heart Function Assessment (NYHA) Classification

**DOI:** 10.1371/journal.pone.0104312

**Published:** 2014-09-02

**Authors:** Shuai Zhang, Zhi-gang Yang, Jia-yu Sun, Ling-yi Wen, Hua-yan Xu, Ge Zhang, Ying-kun Guo

**Affiliations:** 1 Department of Radiology, National Key Laboratory of Biotherapy, West China Hospital, Sichuan University, Chengdu, China; 2 Department of Radiology, West China Second University Hospital, Sichuan University, Chengdu, China; Loyola University Chicago, United States of America

## Abstract

**Purpose:**

To determine whether 3.0-T magnetic resonance imaging (MRI) could assess right ventricular (RV) function in patients with hypertrophic cardiomyopathy (HCM), and if this assessment is correlated with the New York Heart Function Assessment (NYHA) classification.

**Materials and Methods:**

Forty-six patients with HCM and 23 normal individuals were recruited. Left and right ventricular function parameters including end-diastolic and end-systolic volumes (EDV, ESV), stroke volume (SV) and ejection fraction (EF) and dimensions were measured and compared using 3.0-T MRI. RV function parameters between HCM patients and controls were compared using independent sample t tests. A one way ANOVA test with Bonferroni correction was used to determine significant differences among different NYHA groups. Receiver operating characteristic analyses calculated the sensitivity and specificity of RV dysfunction on MRI for the prediction of HCM severity.

**Results:**

Statistical analysis revealed significant differences of left ventricular (LV) and RV volumetric values and masses between the HCM patients and controls (all p<0.05). Within the HCM group, the simultaneously decreased maximum RVEDD correlated well with the LVEDD (r = 0.53; p<0.001). The function and dimension parameters among Class I to III were not determined to be significantly different (all p>0.05). However, significant differences between the Class IV and I-III groups (all P<0.0167) indicated that the diastolic and systolic function in both the RV and LV were impaired in Class IV patients. ROC analyses identified the EDV, ESV and EDD of both the LV and RV with a high sensitivity cutoff value to predict the HCM patients with severe heart failure (Class IV) with high sensitivity and specificity.

**Conclusions:**

RV involvements were comparable to those of LV global function impairments in patients with HCM. The presence of RV dysfunction and decreased dimension on the MRI helped to predict the severe symptomatic HCM with high sensitivity and specificity.

## Introduction

Hypertrophic cardiomyopathy (HCM) is the most prevalent, heritable cardiovascular disease with a prevalence rate of 1 in 500 [1]. The clinical manifestation of HCM is characterized by unexplained left ventricular (LV) hypertrophy, various patterns of wall thickening and the impairment of cardiac function to some extent [2]. Currently, most clinical evaluations focus on LV function, ignoring the right ventricular (RV) function [3], [4]. In our previous study, we evaluated the regional myocardial microvascular dysfunction differences in hypertrophic cardiomyopathy (HCM) patients with or without LV outflow tract obstruction with first-pass perfusion CMR imaging [5]. Moreover, RV dysfunction is of interest as a strong indicator of cardiovascular morbidity and mortality in patients with heart failure. In various clinical settings, right heart failure may be caused by chronic pressure overload that is prone to be ignored for prolonged periods [6]–[8]. Thus, the evaluation of RV dysfunction is necessary in patients with HCM.

Currently, echocardiography is the most common imaging modality used to assess cardiac function. However, two-dimensional echocardiography has several limitations including operator dependence, inadequate endocardial border discrimination and geometric assumptions. Because the RV has complex geometry, it is difficult to accurately measure RV function parameters using conventional imaging modalities [9], [10]. In a spectrum of cardiac diseases involving the RV, magnetic resonance imaging (MRI) is generally accepted as the standard of reference for RV structure and function because of its high spatial and temporal resolution [10]–[15]. The clinical values of the RV parameters in patients with heart failure are well documented, and to the best of our knowledge, data related to RV function and dimension in patients with HCM correlated with the New York Heart Function Assessment (NYHA) classification needs be further determined.

## Materials and Methods

### 1. Ethics Statement

For its retrospective characteristic of this study, a application for exemption of patients' informed consents was approved by the Institutional Review Board (IRB), and we pledged to abide by the declaration of Helsinki (2000 EDITION) in accordance with the relevant medical research rules of China in the study. In addition, no intervention were given in participations with strict secrecy for personal information and privacy. IRB at West China Hospital of Sichuan University approved this study.

### 2. Study population

Patients with HCM that underwent MRI at our hospital from February 2011 to October 2012 were initially enrolled in the study. After 2-dimentional echocardiography examination and clinical assessment, diagnosis of HCM was established based on the practice guidelines of the American College of Cardiology/European Society of Cardiology [16]. The exclusion criteria for the HCM group included moderate degree of mitral regurgitation or tricuspid regurgitation, left or right bundle branch block, coronary artery disease and previous treatments such as myomectomy or ablation. Forty-six patients with HCM (mean age, 51.17±15.18 years; 21 men) were included in the study. Twenty-three normal individuals (mean age, 45.65±14.89 years; 10 men) who underwent MRI during the same period were selected as the normal control group, on the basis of family histories or clinical suspicion of either structural cardiovascular disease or anomalous coronary origins served as controls. All controls were not family members of HCM patients. The exclusion criteria were structural heart disease measured by CMR and 2D TTE, electrocardiogram abnormalities, or clinical histories of cardiovascular diseases.

### 3. Cardiac MRI

Prior to MRI examination, a clinical evaluation and assessment of symptoms using the NYHA classification were conducted by the patient's referring cardiologist. All patients and normal controls were examined using a 3.0-T whole-body scanner (Trio Tim; Siemens Medical Solutions, Erlangen, Germany) in the supine position with a dedicated two-element cardiac phased array coil to assess cardiac function. Electrocardiographic gating and breath holding in expiration were used when feasible. The transverse, coronal and sagittal plane localizing images were acquired using TrueFISP sequence (TR/TE 232.56/1.16 ms, flip angle 60°, slice thickness 8 mm, spacing between slices 12 mm, field of view 290×373 mm, matrix size 146×224 mm). Double-angulated long-axis two- and four-chamber images were acquired with a segmented ECG-gated FLASH 2D cine sequence (TR/TE 37.66/1.2 ms, flip angle 50°, slice thickness 8 mm, field of view 278×330 mm, matrix size 130×192). For functional measurement, the short axis images from the base to the apex were acquired via 8–12 images with turboflash sequences (repetition time, 154.38; echo time, 1.07; inversion time, 90; flip angle, 10°; acquisition matrix, 106×192; field of view, 270×460 mm; slice thickness, 10 mm; spacing between slices, 0 mm). Forty-three patients in this study had underwent first-pass perfusion CMR imaging to evaluate the regional myocardial microvascular dysfunction differences in hypertrophic cardiomyopathy (HCM) patients with or without left ventricular outflow tract obstruction [5].

### 4. Image analysis

**Figure 1:**
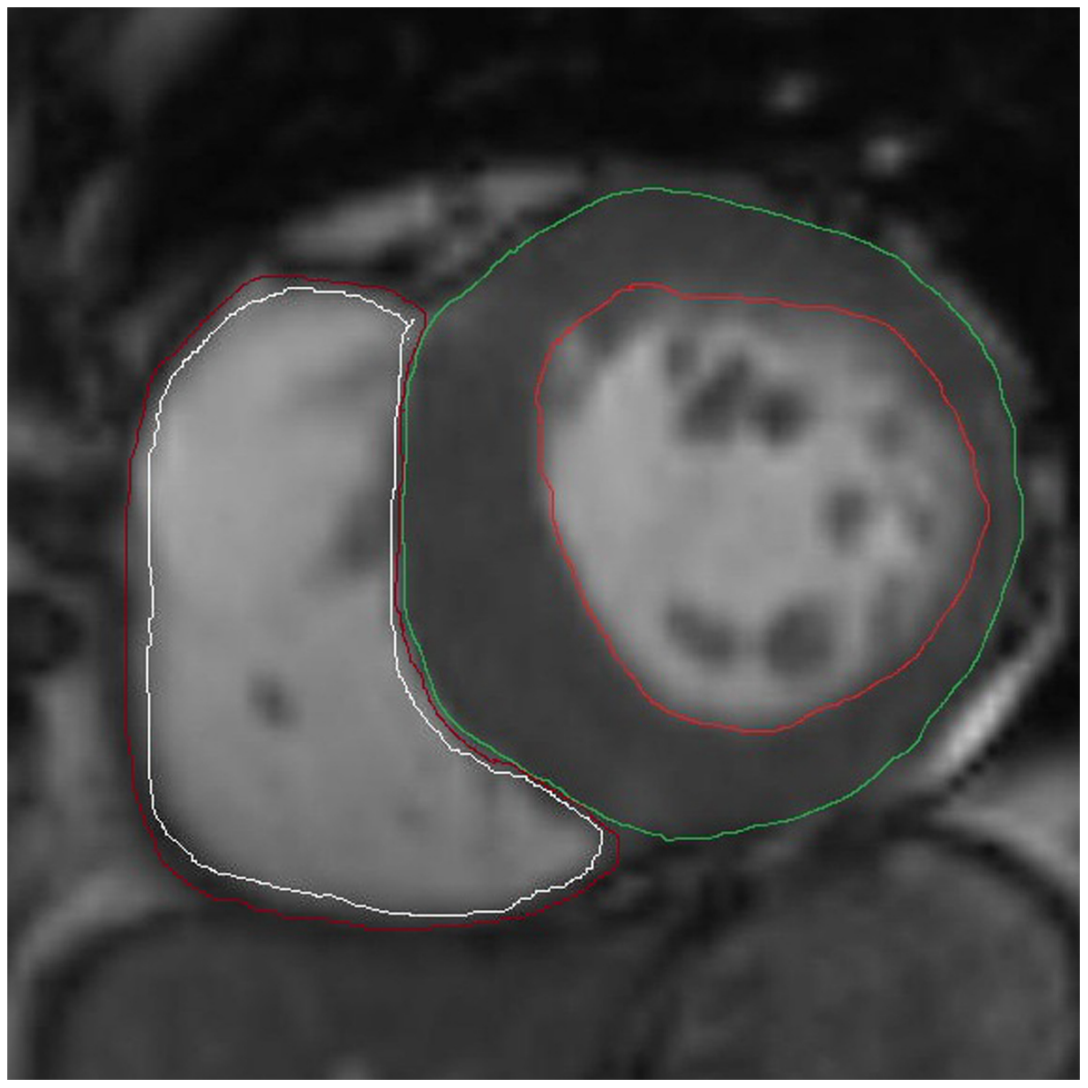
Cardiac images show an example of left and right ventricular function data obtained from the patient at end-diastole phase using MRI.

**Figure 2:**
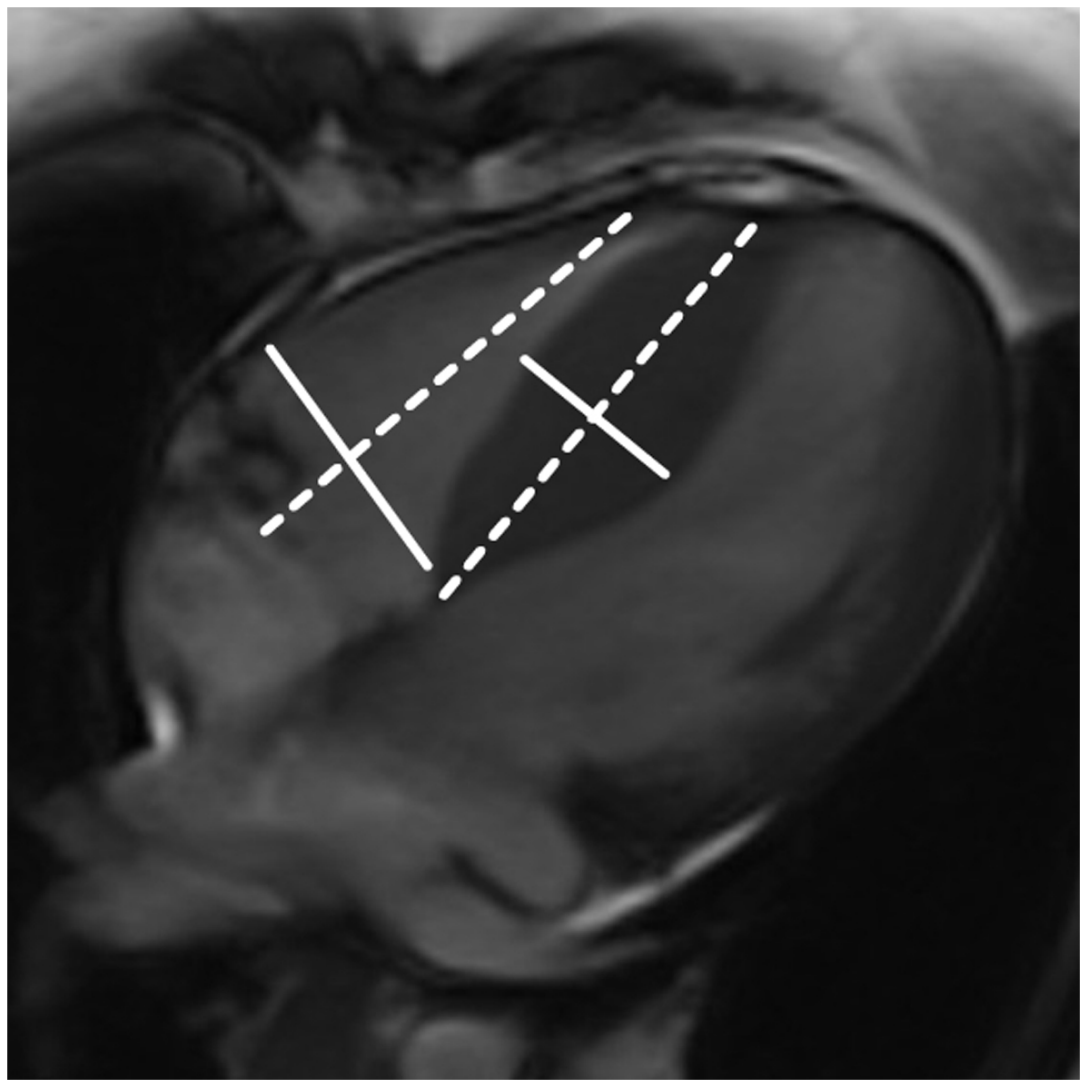
Measurement of the right ventricular and IVS dimensions from 4-CH cardiac views in patients with HCM.

Cine MRI data were analyzed offline with commercial software (Argus, Siemens Medical Solution, Erlangen, Germany) by an experienced observer without access to any patient information. In each short-axis slice, the myocardial contours were delineated on images of the end-diastolic phases, which were visually identified as the largest chamber areas, and the end-systolic phases, which were visually identified as the smallest chamber areas, to obtain the function parameters (EDV, ESV, SV and EF). Papillary muscles and moderator bands were carefully assigned to the lumen of the ventricle (**Fig. 1**). Maximum LV and RV dimensions (LVEDD and RVEDD) and the thickness of the interventricular septum (IVS) (i.e., the maximal distance between the ventricular endocardium and the interventricular septum, perpendicular to the long axis) were measured with callipers using the four-chamber views (Fig. 2).

### 5. Variability analysis

To determine the intra-observer variability of MRI assessment of RV function, one experienced radiologist first measured all RV function values, and the same investigator repeated the measurement in 30 randomly selected patients one month later. Furthermore, to determine the inter-observer variability, a second experienced investigator re-analyzed the measurements of those 30 patients without knowledge of the results of the first investigator. Variability values in each patient were averaged over the entire patient group, and these values are presented as the mean ± standard deviation.

### 6. Statistical analysis

Statistical analysis was performed with commercially available software (SPSS for Windows, version 19.0; SPSS Inc., Chicago, IL). Continuous variables were presented as the mean ± standard deviation (SD). All the function parameters were standardized with body surface area (BSA). The differences in function and size parameters between the HCM and control groups were evaluated using an independent t test. The Kruskal–Wallis and Wilcoxon tests for unpaired samples were used to examine the differences in functional parameters among the NYHA classification groups. Bonferroni correction was performed for multiple pairwise comparisons by adjusting the significance level (α).The Spearman correlation test was used to examine the relationship between the functional parameters and the NYHA classification. Receiver operating characteristic (ROC) analysis was used to predict the NYHA class. For all statistical testing, a two-tailed *P* value of <0.05 was considered statistically significant.

## Results

### 1. Patient characteristics

A total of 46 HCM patients and 23 healthy controls were eligible for the study (**Table 1**). There were no significant differences in age, sex, weight, height and BSA between the two groups (all *p*>0.05). According to NYHA classifications, 14 patients (30.43%) had no symptoms (NYHA class I), 15 patients (32.61%) were mildly symptomatic (NYHA class II), 11 (23.91%) were moderately symptomatic (class III) and 6 (13.04%) were severely symptomatic (class IV). The condition of the patients was stable during the examination period. All imaging examinations were performed successfully in all patients without any complications. Good quality images from each imaging technique were obtained for function analyses. Overall MRI time was 20–30 min, and it took approximately 20 min to obtain all the measurements.

**Table 1:** Characteristics of HCM patients and normal individuals.

	HCM (N = 46)	Health controls (N = 23)	*p* value
Age, y	51.17±15.18	45.65±14.89	.157
Male gender	45.7%	43.5%	.864
Angina pectoris	43%	-	-
Dyspnea	3%	-	-
Syncope	22%	-	-
Current smoking	32.6%	30.4%	.855
Weight, kg	60.09±10.98	59.59±9.72	.854
Height, cm	159.11±8.42	162.48±7.94	.115
Body surface area, m^2^	1.71±0.17	1.72±0.15	.737
NYHA, I/II/III/IV/	14/15/11/6	-	-

Data given as the mean ±SD.

### 2. Comparison between the HCM patients and normal controls

**Figure 3:**
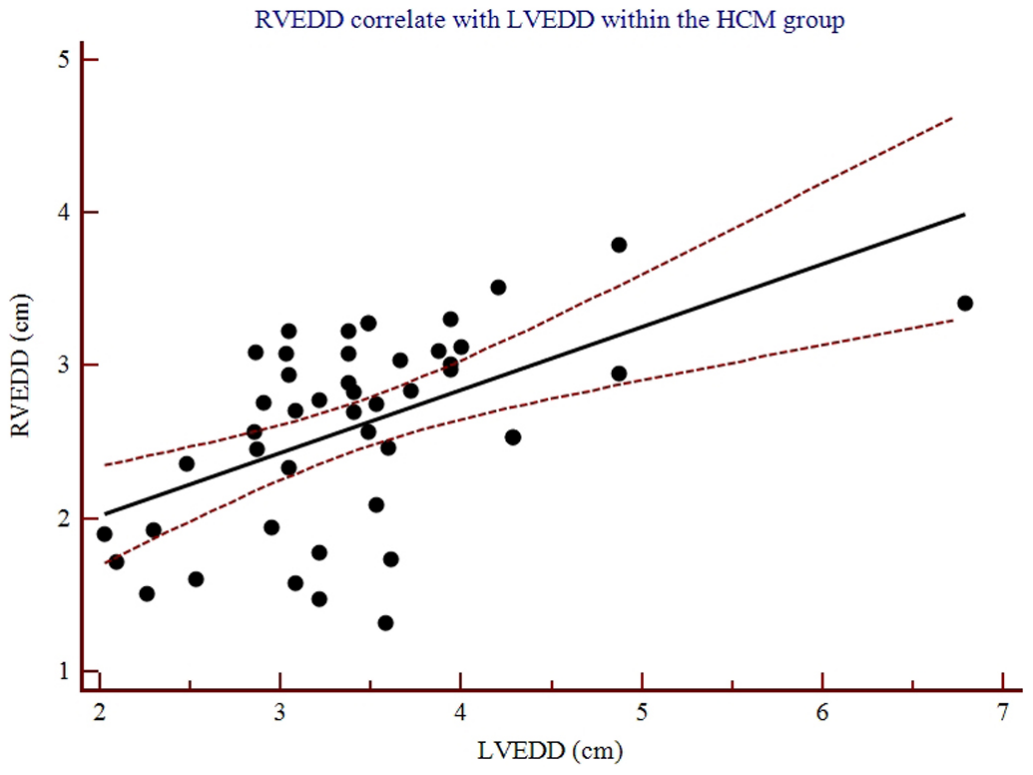
Scatterplots show the close relationship between RVEDD and LVEDD measuring with cardiac MRI (r = 0.53, 95% CI: 0.39, 0.71; p<0.001).

The mean values of function and size parameters on the MRI in 46 patients with HCM and 23 normal controls are summarized in **Table 2**. Statistical analysis revealed significant differences of LV and RV volumetric values and masses between the HCM patient and controls (all p<0.05). No significant difference in the EF values (p = 0.251, 0.486) was found between the HCM patients and healthy subjects. Accordingly, the decreased maximum LV and RV dimensions (LVEDD and RVEDD) and the thickness of the interventricular septum (IVS) were revealed to be significantly different between the HCM patients and the controls (all p<0.05). Within the HCM group, the simultaneously decreased maximum RVEDD correlated well with the LVEDD (r = 0.53, 95% CI: 0.39, 0.71; p<0.001) (Fig. 3).

**Table 2:** RV and LV function and size parameters of HCM patients and normal individuals.

Variable	HCM (n = 46)	Health controls (n = 23)	*p* value
RVEDV, ml/m^2^	51.45±13.58	65.31±11.39	.000
RVESV, ml/m^2^	24.43±9.92	31.26±7.34	.003
RVSV, ml/m^2^	27.32±8.42	36.83±6.33	.000
RVEF, %	53.58 ±10.67	52.15±6.21	.486
RVMASS, g/m^2^	31.39±6.73	20,16±7.66	.000
RVEDD, cm	2.61±0.62	3.28±0.46	.000
IVST, cm	2.34±0.53	0.90±0.13	.000
LVEDV, ml/m^2^	75.30±26.73	86.13±10.36	.019
LVESV, ml/m^2^	29.94±25.36	39.54±6.43	.028
LVSV, ml/m^2^	45.38±14.31	51.60±6.28	.015
LVEF, %	62.59±13.76	59.99±4.62	.251
LVMASS, g/m^2^	119.33±37.53	48.59±10.58	.000
LVEDD, cm	3.43±0.81	4.41±0.35	.000

Data given as the mean ±SD.

RVEF, right ventricular ejection fraction; RVEDV, right ventricular end diastolic volume; RVESV, right ventricular end systolic volume; RVSV, right ventricular stroke volume; IVST, interventricular septal thickness; RVEDD, right ventricular end diastolic diameter.

### 3. The function and dimension parameters of HCM patients correlated with NYHA classifications

**Figure 4:**
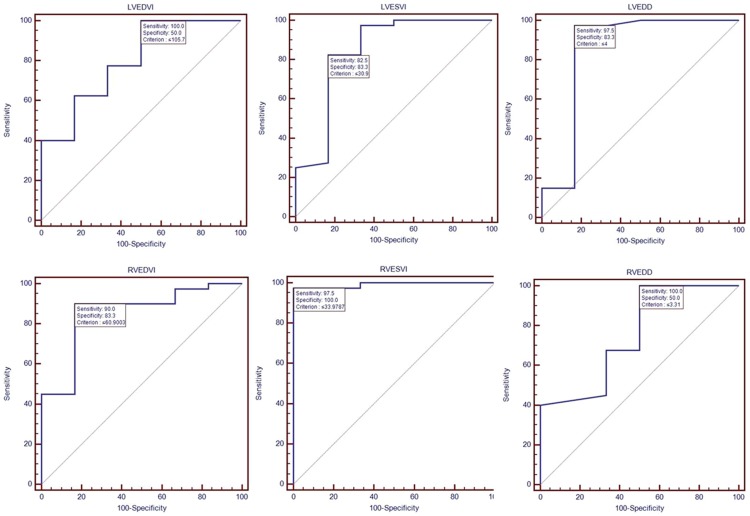
With ROC analyses, the sensitivity and specificity of RV and LV dysfunction and dimension on cardiac MRI for predicting HCM patients with severe heart failure (Class IV) were high.

For all 46 patients, the mean values of function and size parameters of each subgroup in the different NYHA classifications were calculated and summarized in T**able 3**. The Spearman correlation test indicated a weak correlation between the functional and size parameters and the different NYHA classifications (r = 0.059 to 0.378; all p<0.05). After Bonferroni correction, the functional and size parameters among Class I to III were found not to be significantly different (all p>0.05). However, significant differences between the Class IV and I–III groups (all *P*<0.0167) indicated that thediastolic and systolic function in both the RV and LV were impaired in Class IV patients. Although no significant differences of EF values of both ventricles were found between the HCM patients and healthy subjects, the EFs between the class IV and class I–III patients were significant difference (p<0.05), and similar results were found between the class IV patients and the controls (p<0.05). Similarly, dimensional limitations (LVEDD and RVEDD) were revealed in those Class IV patients (p<0.001), but for IVS thickness (p>0.05). ROC analyses identified the EDV, ESV and EDD of both the LV and RV with a high sensitivity cutoff value, as predictors of the HCM patients with severe heart failure (Class IV) with high sensitivity and specificity (**Fig. 4**).

**Table 3:** Function and size parameters in different NYHA classifications.

	Class I	Class II	Class III	Class IV	Spearman coefficient
RVEF, %	56.01±9.03	57.93±7.93	53.67±7.67	36.83±10.40*	−.367
RVEDV, ml/m^2^	51.95±12.55	51.67±13.19	43.21±8.67	64.85±15.69*	.027
RVESV, ml/m^2^	22.73±7.09	21.73±7.02	19.98±4.95	43.29±8.20*	.240
RVEDD, cm	2.46±0.60	2.35±0.64	2.88±0.45	3.13±0.55*	.378
IVST, cm	2.36±0.41	2.47±0.61	2.01±0.49	2.47±0.53	−.059
LVEF, %	65.36±8.96	65.93±7.01	65.56±11.94	42.35±22.50*	−.191
LVEDV, ml/m^2^	67.81±18.61	76.74±17.00	63.93±18.04	110.00±46.67*	.133
LVESV, ml/m^2^	22.53±6.09	26.03±7.05	22.43±11.88	70.77±54.42*	.207
LVEDD, cm	3.22±0.62	3.21±0.50	3.34±0.50	4.64±1.28*	.295

Data given as the mean ±SD. *, *p*<0.05, compared to Class I, Class II and Class III.

### 4. Variability of the 3.0-T MRI measurements

The inter- and intra-observer variability for RV functional measurements of the MRI were calculated and are summarized in **Table 4**. Inter- and intra-observer variability of RV function from the MRI ranged from 4.9% to 8.5% and 4.0% to 9.5%, respectively.

**Table 4:** Interobserver and intraobserver variability of RV functional parameters.

	RVEF	RVEDV	RVESV	RVSV	RVMASS
Interobserver	9.5%±4.4	4.9%±2.7	7.1%±4.6	8.2%±5.0	6.4%±3.7
Intraobserver	8.5%±4.2	4.0%±2.2	5.7%±4.1	8.2%±4.3	5.7%±3.4

## Discussion

This study demonstrated that the RV involvements were comparable to those of LV global function impairments in patients with HCM. Similarly, the decreased dimension of the maximum RVEDD correlated well with the LVEDD. No significant relation was evident between the global function and morphologic dimensional index in the Class I to III patients with HCM. However, the presence of RV dysfunction and decreased RVEDD on the MR imaging helped predict the severity of Class IV (NYHA) in HCM, as well as the corresponding LV parameters.

Based on previous reports, RV dysfunction plays a vital role in determining the clinical outcomes of patients with heart failure [6]–[8]. Therefore, evaluation of the RV function is clinically important for the diagnosis, therapy and follow-up of a broad spectrum of cardiac and pulmonary diseases [10]–[12]. As a reference standard, cardiac MRI allows quantification of myocardial thickness, function, and left ventricular outflow tract obstruction in HCM [14], [17]–[19]. Thus, the function and size of RV in patients with HCM needs be further determined with cardiac MRI because RV dysfunction might play a key role in the outcome of patients with HCM [20].

In the present study, we found that the RV involvements were comparable to those of LV global function impairments in patients with HCM. Similarly, previous studies demonstrated that the magnitude of maximum RV wall thickness and mass correlated significantly with LV wall [18]. Except for global function impairment, Maron and their colleagues' [18] found that abnormalities in the RV wall thickness and mass are common in HCM patients. The reasons can be explained that ventricular inter-dependence may play an important component in RV involvement. Ventricular inter-dependence means that the size, shape and compliance of one ventricle may affect other ventricles through direct mechanical interactions [21]. The shifts of the LV and the inter-ventricular septum in HCM patients alter the RV geometry, causing states of decreased RV volume and low RV cardiac output states. Unlike Suzuki's study [17], no significant differences in the RVEF were observed in our study when compared with the normal controls. In contrast to their study, moderate and severe symptoms (class III and IV) accounted for a large proportion of our study population, and their RVEF values had a declining trend during the present study.

Additionally, the current study underlines the importance of the RV diastolic dysfunction in HCM patients. Mörner et al [22] have indicated that the LV diastolic dysfunction seems to be an early phenomenon explaining much of the symptomatology in the disease, although LV systolic function is not a primary problem. Additionally, other studies have suggested that the RV diastolic dysfunction assessment cannot be disregarded in clinical managements because the RV diastolic function indices can be regarded as markers in the prognosis of HCM patients [19], [20]. Although the RV dimension is a standard component of the echocardiographic report, it is of clinical importance to realize that the RV dimension carries important clinical and prognostic information in patients with HCM due to the decreased dimension of the maximum RVEDD strongly correlated with the LVEDD [12]. In HCM patients, chamber stiffness can be increased by the increase in muscle mass and muscle stiffness caused by myocardial fibrosis. This increased chamber stiffness can result in an increased diastolic pressure, and consequently, impaired diastolic functioning resulting in a decrease of both the RVEDD and LVEDD [21].

Unlike previous reports [17], [18], [22], thirteen percent HCM patients with severely symptomatic (NYHA, Class IV) were enrolled in our series. Although no relation was evident among the global function and morphologic dimensional index and the Class I to III patients with HCM, significant differences between the Class IV and I–III groups indicated that diastolic and systolic function in both the RV and LV were impaired in Class IV patients. This study demonstrated that the presence of RV dysfunction and decreased RVEDD on the cardiac MRI was useful for the prediction of severe symptomatic HCM (Class IV, NYHA). For example, sensitivity and specificity values of RVEDV smaller than 60.9 ml/m2 on cardiac MRI for the prediction of Class IV in HCM were 90.0% and 83.3%, respectively. When compared with RVEDV, LEVDV obtained higher sensitivity (100%), but lower unsatisfactory specificity (50%). As shown in the present data, moreover, the presence of RV dysfunction and decreased RVEDD on the MR imaging helped predict the severity of Class IV (NYHA) in HCM patients, as well as those LV parameters.

We acknowledge the following limitations of our study. Until now, right ventricular hypertrophy and scarring in HCM patients is not widely documented [14], [17]–[19], [23]. In most of cohorts, the majority of HCM patients are less symptomatic. In this cohort, however, we described a group of HCM patients that is highly symptomatic; 13% of patients are in NYHA Class IV. This phenomenon might be attributed to selection bias. The important correlation between RV dysfunction and Class IV requires further confirmation. We also did not include objective method (e.g., right heart catheterization) for RV function comparison because MRI is regarded as a reference standard for the quantification of myocardial thickness, function, and ventricular dimension in patients with HCM [7], [13]. Furthermore, the prognostic role of RV dysfunction and dimension measurements on the cardiac MRI in the survival outcomes of HCM patients warrants further investigation by new studies.

In summary, this study demonstrated that RV involvement is comparable to the LV global function impairments in patients with HCM. When HCM patients were severely symptomatic (NYHA, Class IV), the presence of RV dysfunction and decreased dimensions on the MR imaging helped predict the severity of HCM with high sensitivity and specificity.
